# Supplementary material for: Selective sorting of hexane isomers by anion-functionalized metal-organic frameworks with optimal energy regulation
Source: Nat Commun. 2024 Mar 23;15:2620. doi: 10.1038/s41467-024-46738-2 (PMC10960857; doi:10.1038/s41467-024-46738-2)
Supplement: Supplementary file 1 — Supplementary Information [file 41467_2024_46738_MOESM1_ESM.pdf]

Selective sorting of hexane isomers by anion-functionalized metal-organic frameworks with optimal energy regulation

Qingju Wang[1,2], Lifeng Yang[1], Tian Ke[1], Jianbo Hu[1], Xian Suo[2], Xili Cui[1,2,3], Huabin Xing[1,2]

[1]Key Laboratory of Biomass Chemical Engineering of Ministry of Education, College of Chemical and Biological Engineering, Zhejiang University, Hangzhou 310027, China.

[2]Hangzhou Global Scientific and Technological Innovation Center, Zhejiang University, Hangzhou 311215, China.

[3]Shanxi-Zheda Institute of Advanced Materials and Chemical Engineering, Hangzhou 310027, China

*To whom correspondence should be addressed. E-mails: cuixl@zju.edu.cn, xinghb@zju.edu.cn

Supplementary Figures

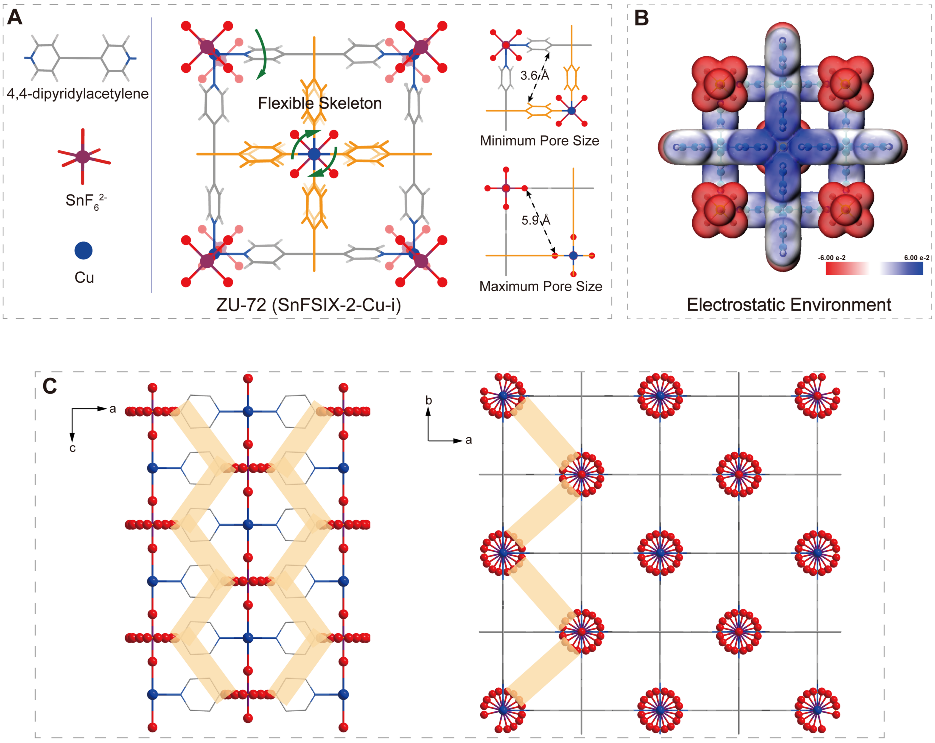

Supplementary Fig. 1. **Crystal Structures of ZU-72**. The building blocks, flexible skeleton and pore sizes of ZU-72 **(a)**. Surface electrostatic potential of ZU-72 (red regions are negative ESP, and blue regions are positive ESP) (**b**). The zigzag binding sites within the frameworks of ZU-72 (**c**).

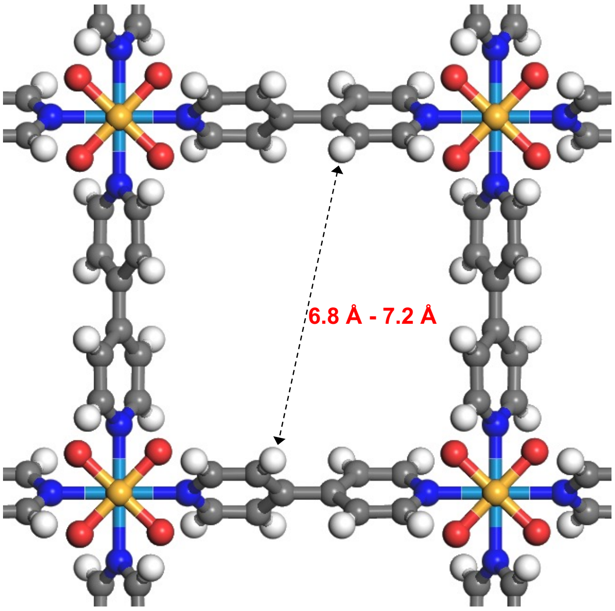

Supplementary Fig. 2. Crystal Structures of SIFSIX-1-Cu. Color code: F, red; Si, light orange; Cu, light blue; C, gray- 40%; H, gray-25%; N, blue.

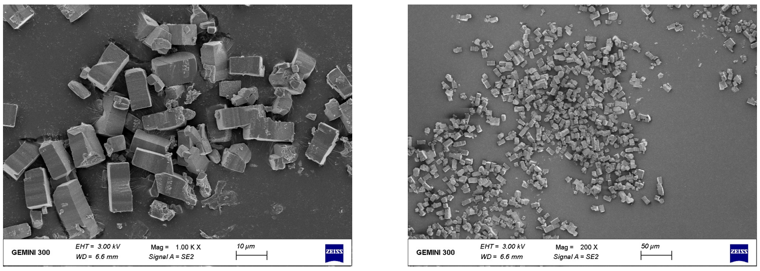

Supplementary Fig. 3. Crystal morphology of SIFSIX-1-Cu.

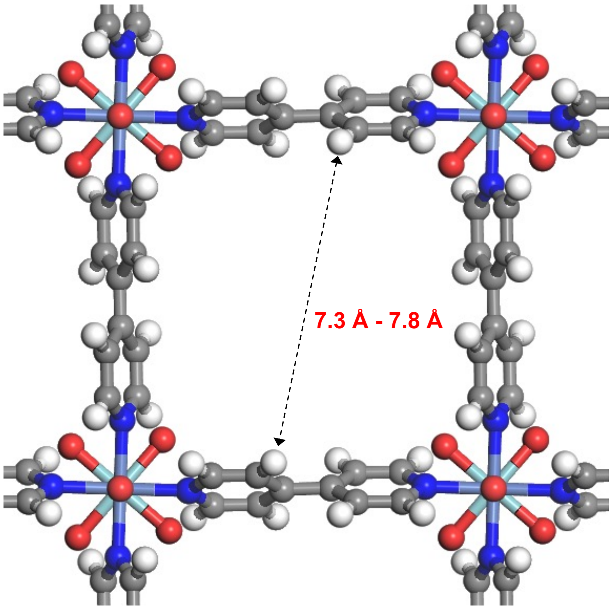

Supplementary Fig. 4. Crystal Structures of ZU-61. Color code: F, red; O, dark red; Nb, cyan; Ni, steel blue; C, gray- 40%; H, gray-25%; N, blue.

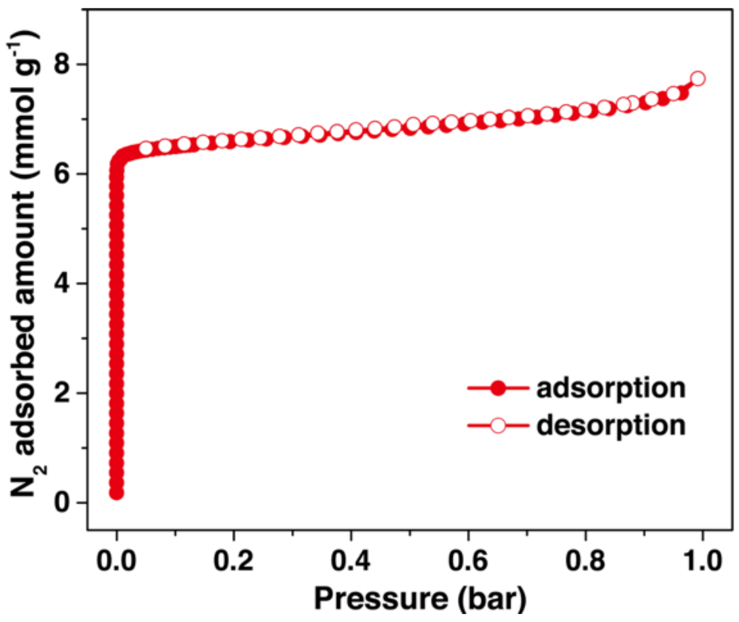

Supplementary Fig. 5. N_2 adsorption isotherm of ZU-72 collected at 77 K.

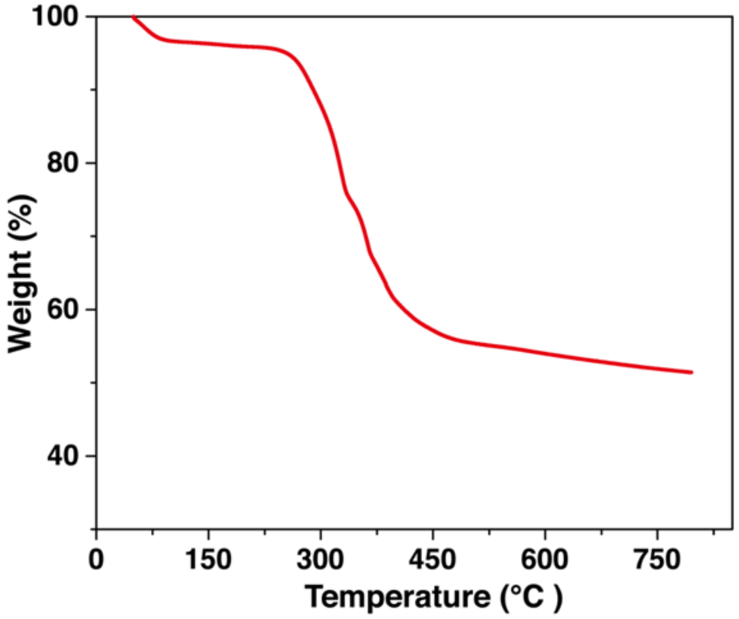

Supplementary Fig. 6. The TGA curve of ZU-72.

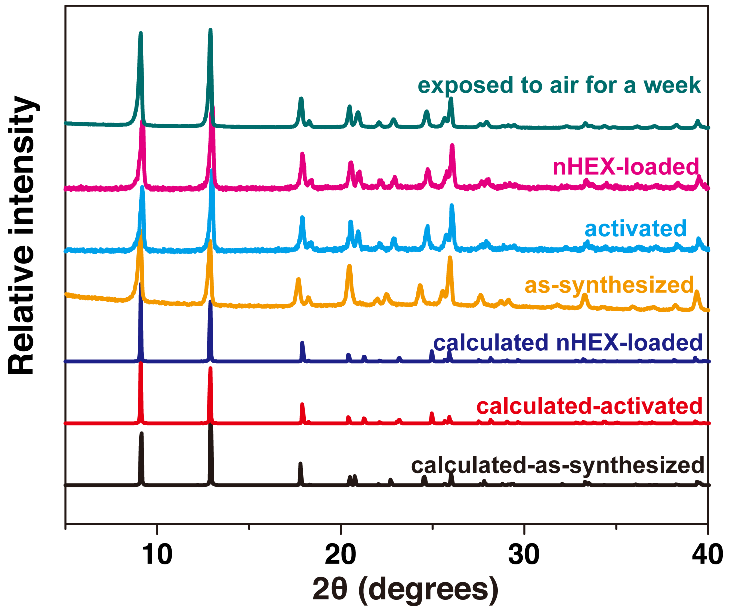

Supplementary Fig. 7. Powder X-ray diffraction patterns of ZU-72.

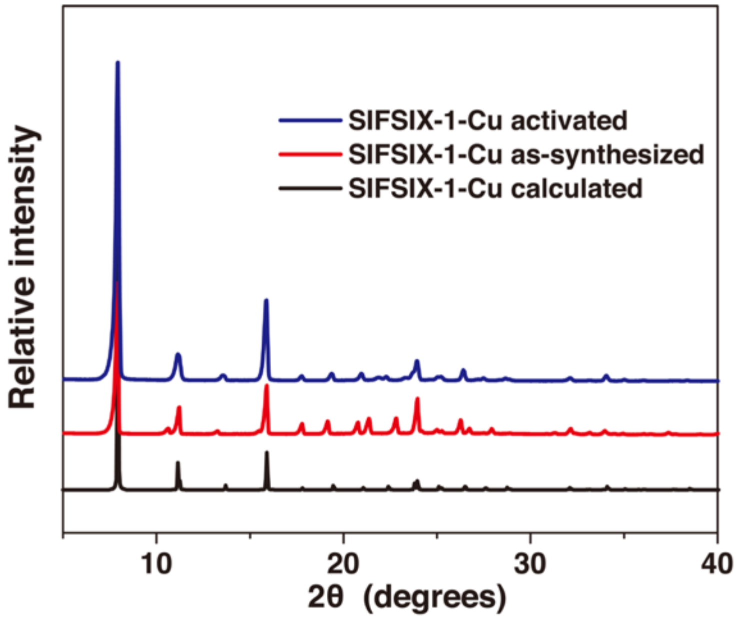

Supplementary Fig. 8. Powder X-ray diffraction patterns of SIFSIX-1-Cu.

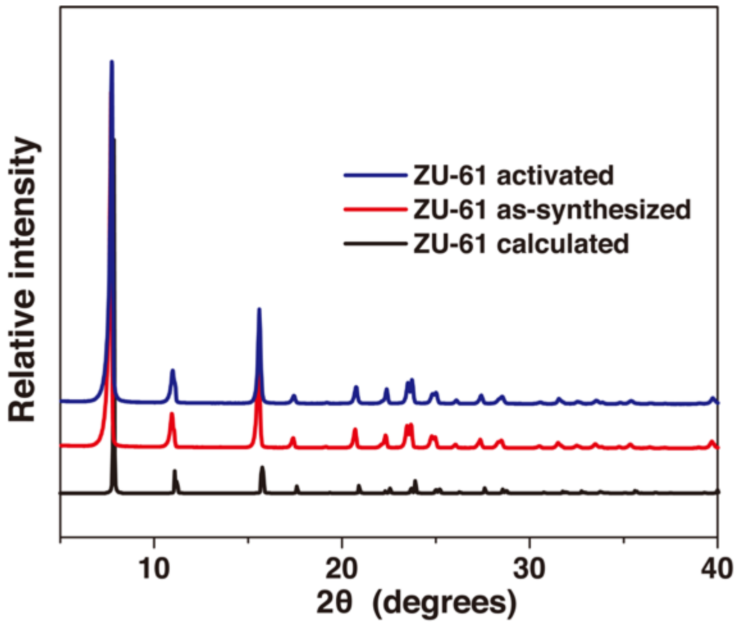

Supplementary Fig. 9. Powder X-ray diffraction patterns of ZU-61.

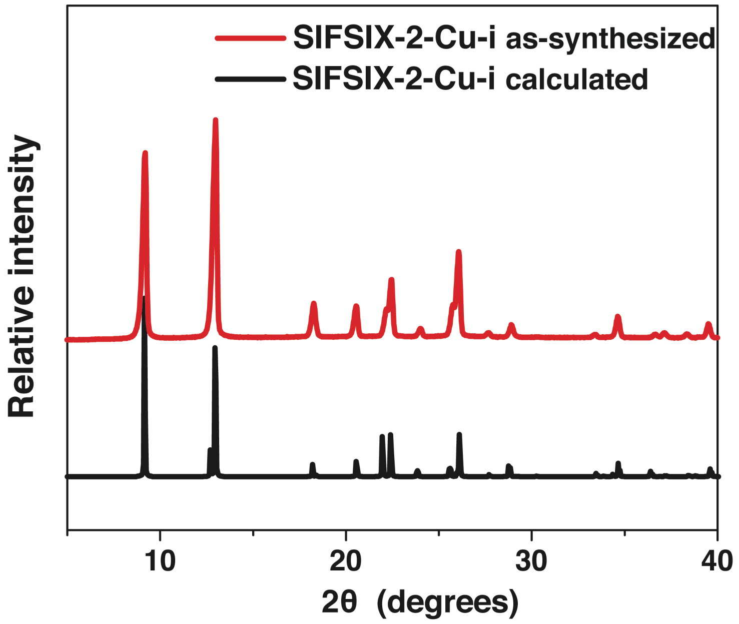

Supplementary Fig. 10. Powder X-ray diffraction patterns of ZU-61.

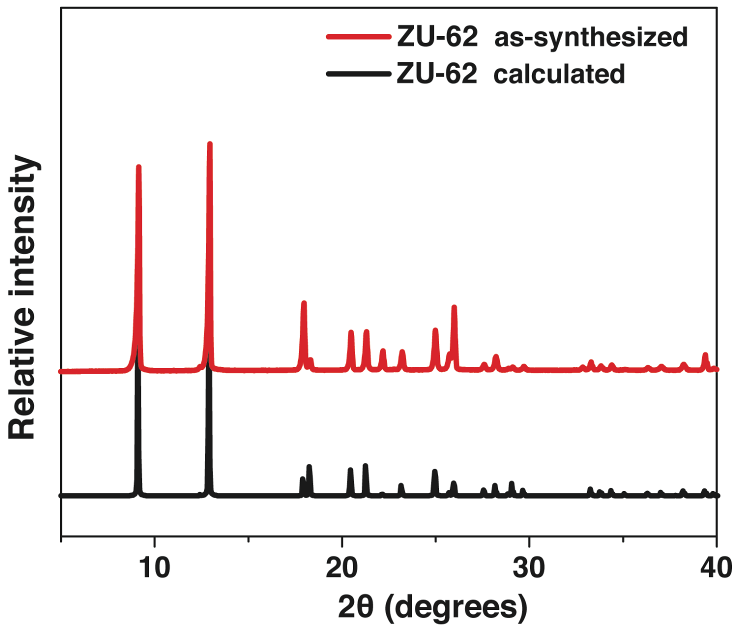

Supplementary Fig. 11. Powder X-ray diffraction patterns of ZU-61.

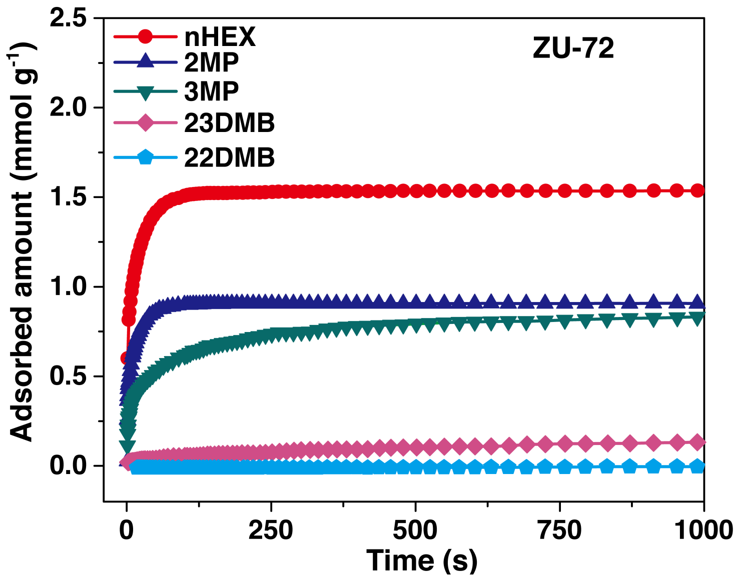

Supplementary Fig. 12. Time-dependent adsorbed amount profiles of n-hexane (nHEX), 2-methylpentane (2MP), 3-methylpentane (3MP), 2,3-dimethylbutane (23DMB) and 2,2-dimethylpentane (22DMB) on ZU-72 at 298 K.

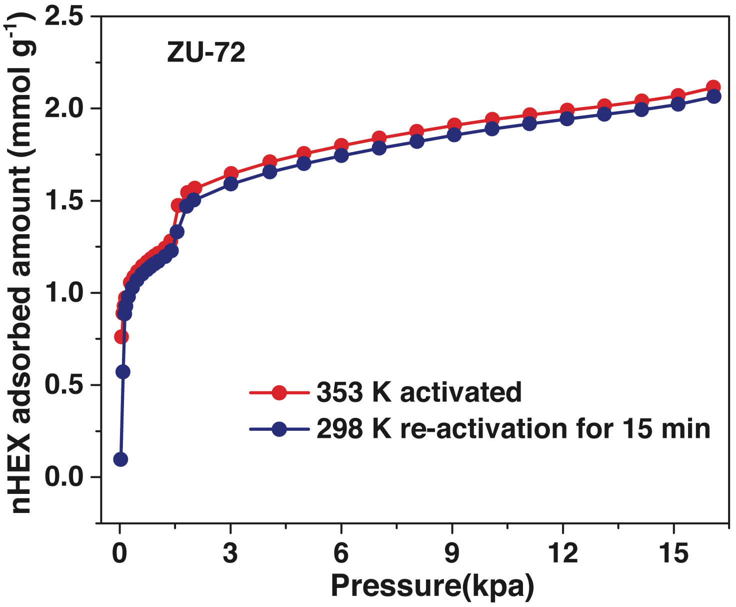

Supplementary Fig. 13. N-hexane (nHEX) adsorption for ZU-72 after activation at 80 °C for 8 hours and re-activation at 25 °C for 15 min.

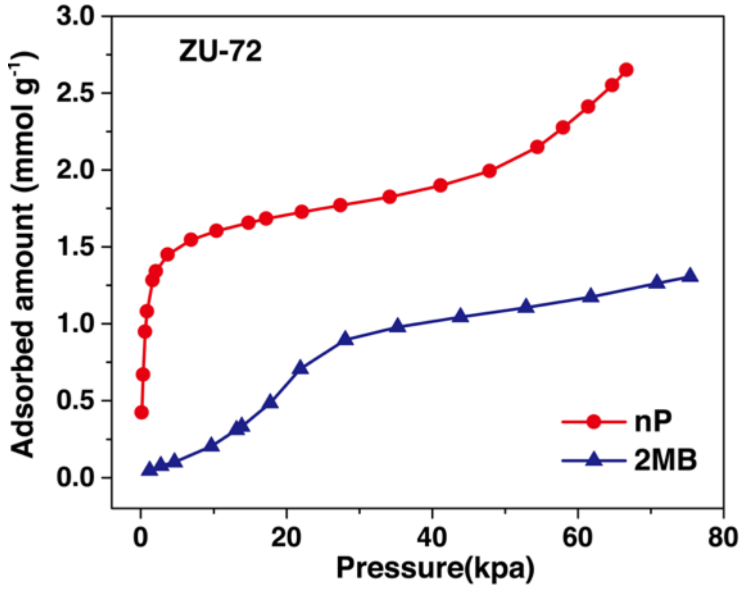

Supplementary Fig. 14. Vapor adsorption isotherms of n-pentane (nP), 2-methylbutane (2MB) on ZU-72 at 298 K.

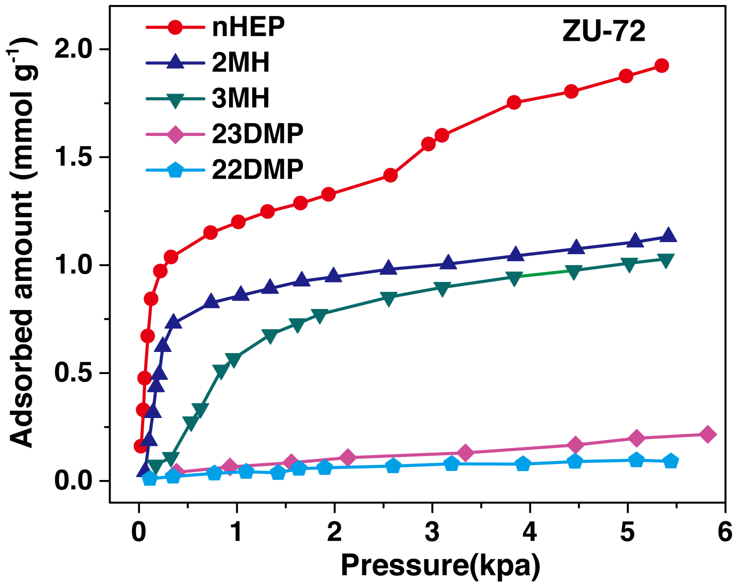

Supplementary Fig. 15. Vapor adsorption isotherms of n-heptane (nHEP), 2-methylhexane (2MH), 3-methylhexane (3MH), 2,3-dimethylpentane (23DMP) and 2,2-dimethylpentane (22DMP) on ZU-72 at 298 K.

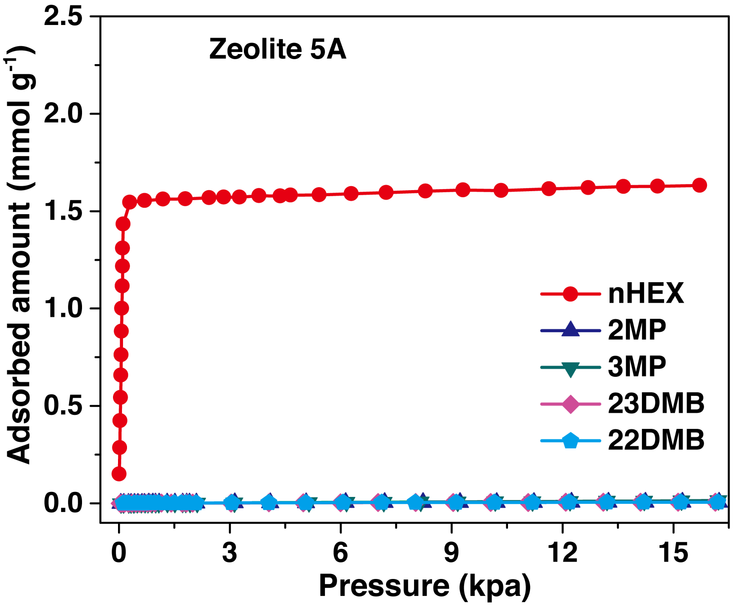

Supplementary Fig. 16. Vapor adsorption isotherms of n-hexane (nHEX), 2-methylpentane (2MP), 3-methylpentane (3MP), 2,3-dimethylbutane (23DMB) and 2,2-dimethylpentane (22DMB) on zeolite 5A at 298 K.

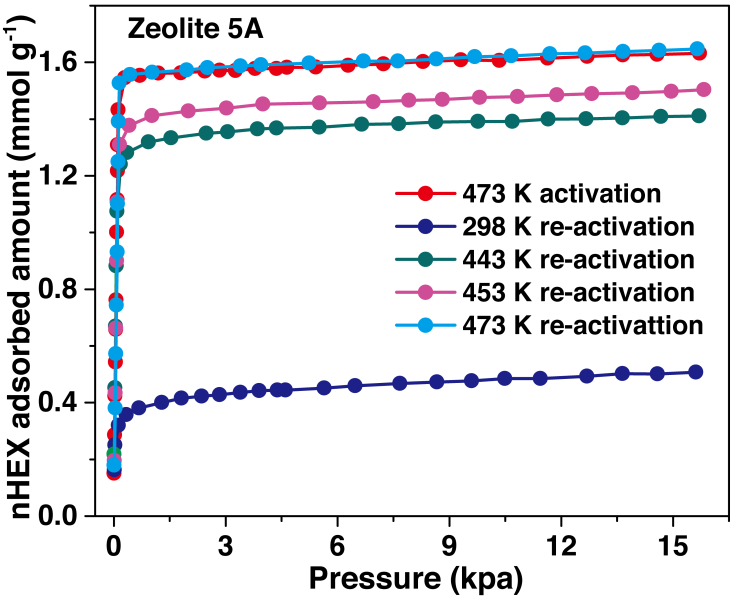

Supplementary Fig. 17. N-hexane (nHEX) adsorption for zeolite 5A after activation at 200 °C for 8 hours and re-activation at 298 K, 443 K, 453 K and 473 K for 8 hours.

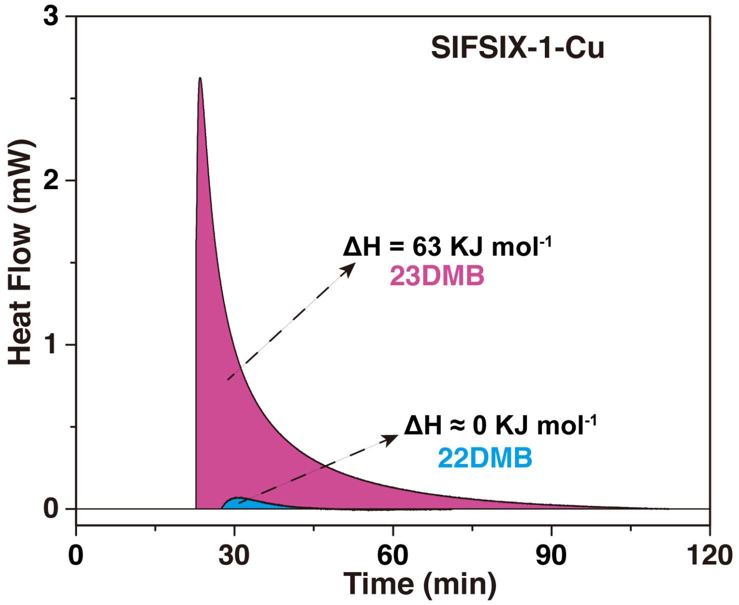

Supplementary Fig. 18. Calorimetric measurements of 2,3-dimethylbutane (23DMB) and 2,2-dimethylbutane (22DMB) adsorption on SIFSIX-1-Cu at 298 K, in which no adsorption heat of 22DMB is detected, in comparison with 63 kJ mol^{-1} for 23DMB.

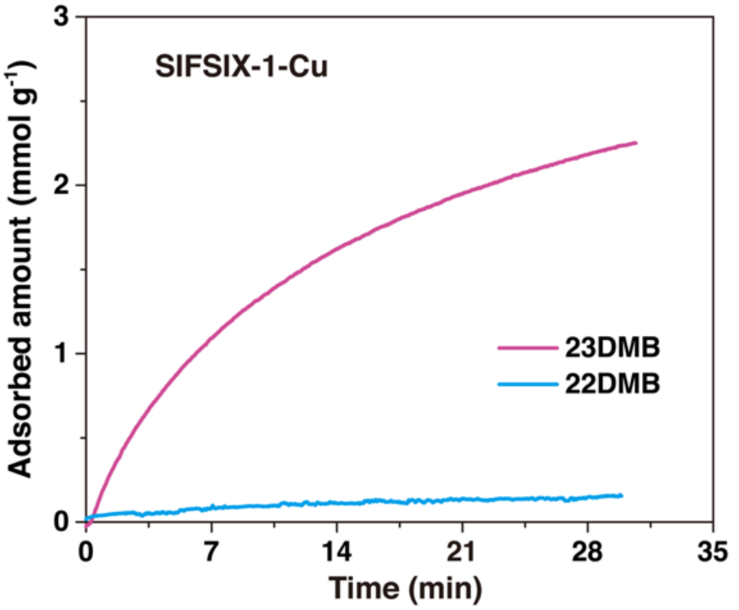

Supplementary Fig. 19. Time-dependent adsorbed amount profiles of 2,3-dimethylbutane (23DMB) and 2,2-dimethylbutane (22DMB) on SIFSIX-1-Cu at 298 K.

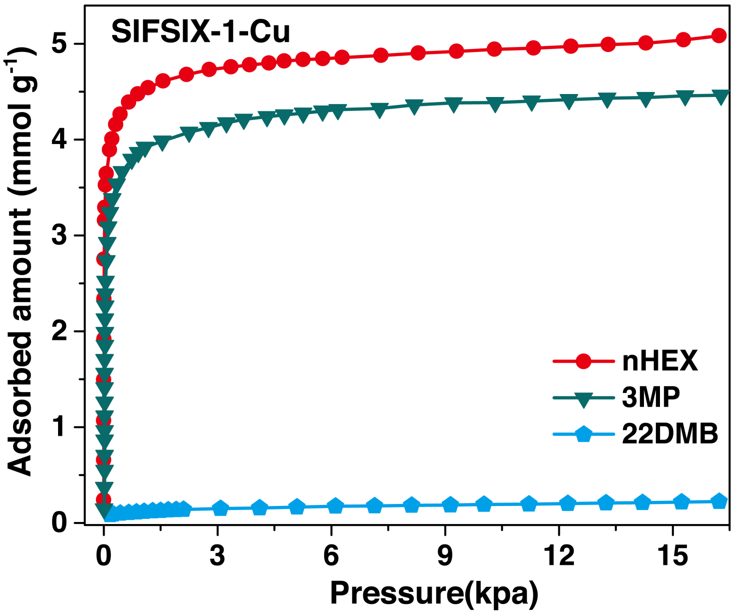

Supplementary Fig. 20. Vapor adsorption isotherms n-hexane (nHEX), 3-methylpentane (3MP) and 2,2-dimethylpentane (22DMB) on on SIFSIX-1-Cu at 298 K.

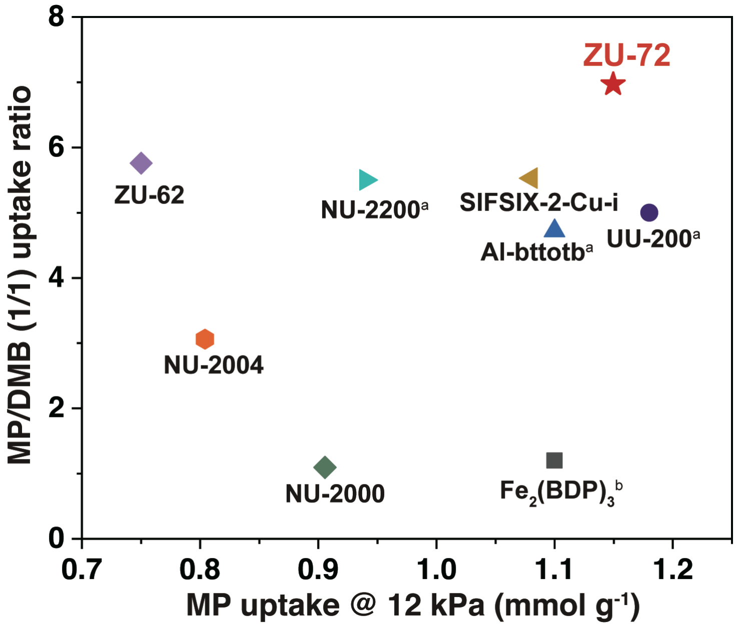

Supplementary Fig. 21. Plots of the MP uptake as a function of the MP/DMB uptake ratio for ZU-72 and other previously reported materials at 298 K and 12 kPa. [a] at 303 K, [b] at 433 K. MP/DMB is defined as (2MP + 3MP)/(23DMB+22DMB).

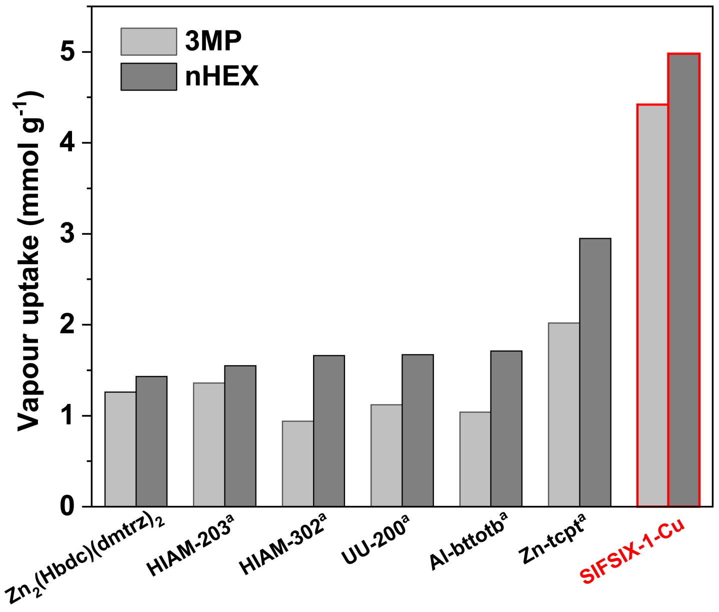

Supplementary Fig. 22. Comparison of n-hexane (nHEX) and 3-methylpentane (3MP) uptake on SIFSIX-1-Cu with other benchmarks which have realized the molecular size sieving of 2,2-dimethylpentane (22DMB) at 12 kPa and 298 K. [a] at 303 K.

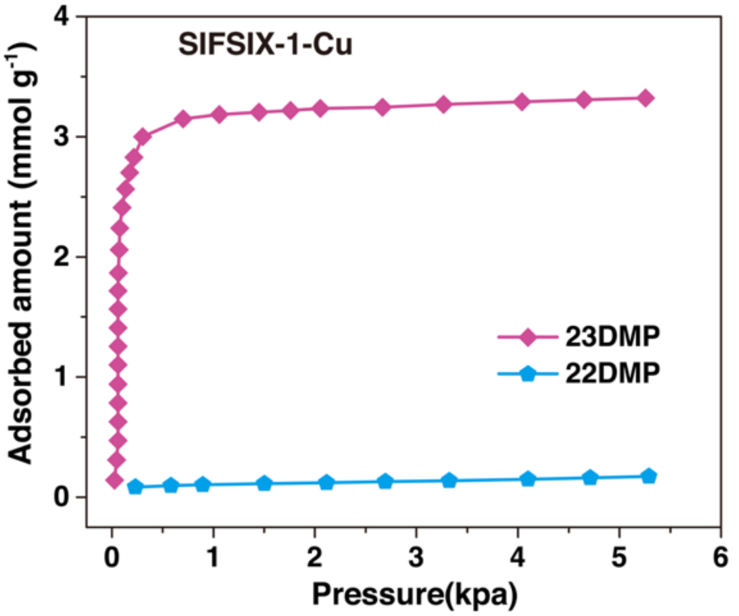

Supplementary Fig. 23. Vapor adsorption isotherms of 2,3-dimethylpentane (23DMP) and 2,2-dimethylpentane (22DMP) on SIFSIX-1-Cu at 298 K.

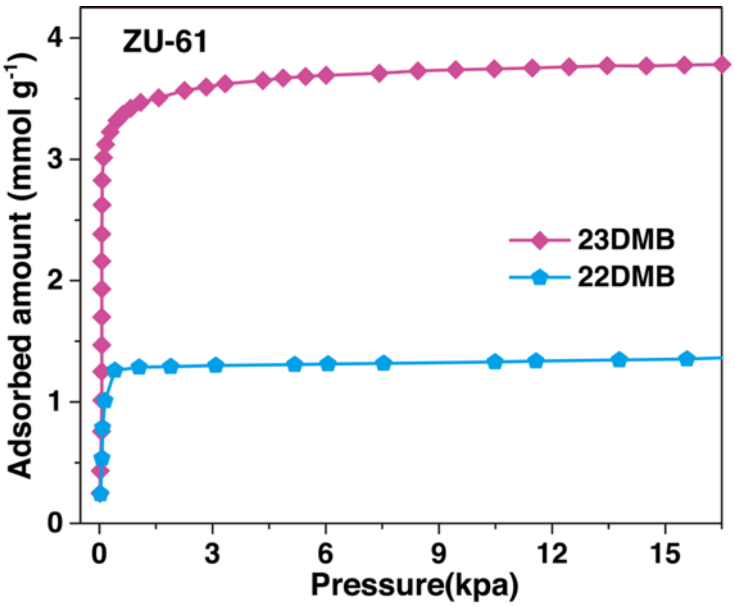

Supplementary Fig. 24. Vapor adsorption isotherms of 2,3-dimethylpentane (23DMB) and 2,2-dimethylpentane (22DMB) on ZU-61 at 298 K.

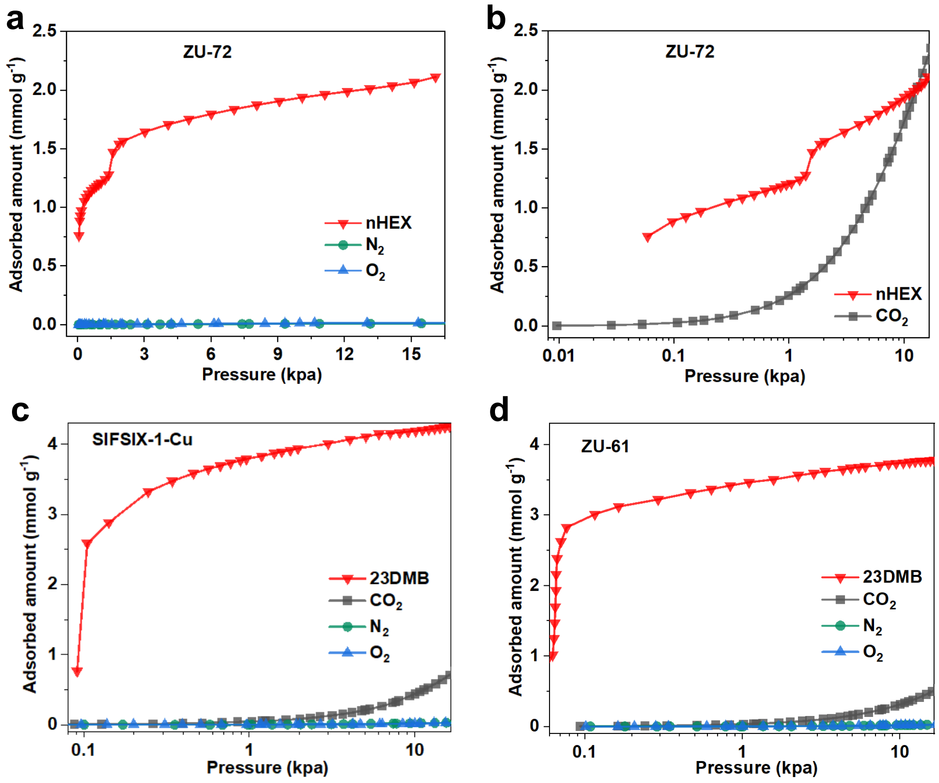

Supplementary Fig. 25. The adsorption isotherms of N_2, O_2, CO_2 and hexane isomers on ZU-72, SIFISX-1-Cu and ZU-61 at 298 K.

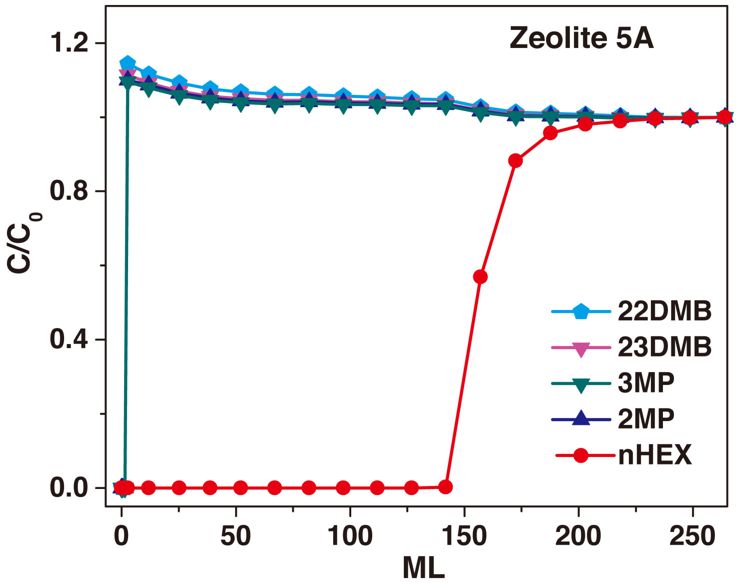

Supplementary Fig. 26. Experimental breakthrough curves for the equimolar mixture of nHEX/2MP/3MP/23DMB/22DMB on zeolite 5A at 298 K. The horizontal axis represents the volume of the mixture of nitrogen gas and hexane isomers.

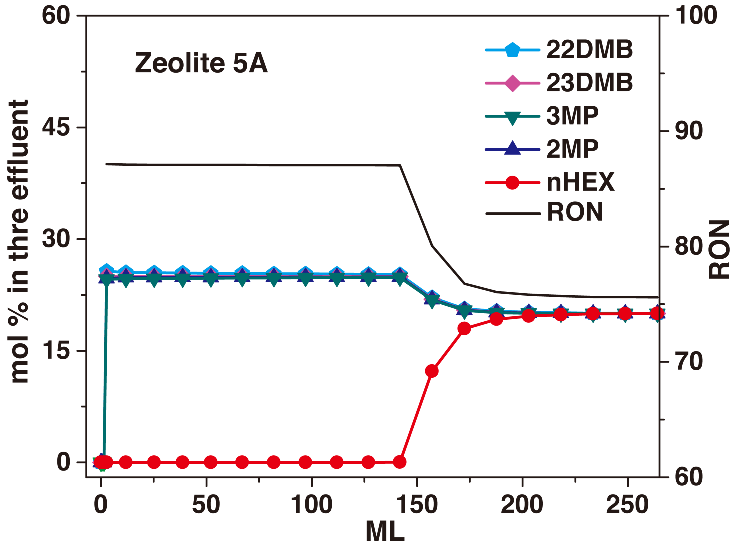

Supplementary Fig. 27. Experimental breakthrough curves for the equimolar mixture of nHEX/2MP/3MP/23DMB/22DMB on zeolite 5A together with the RON calculated form the eluted mixture at 298 K. The horizontal axis represents the volume of the mixture of nitrogen gas and hexane isomers.

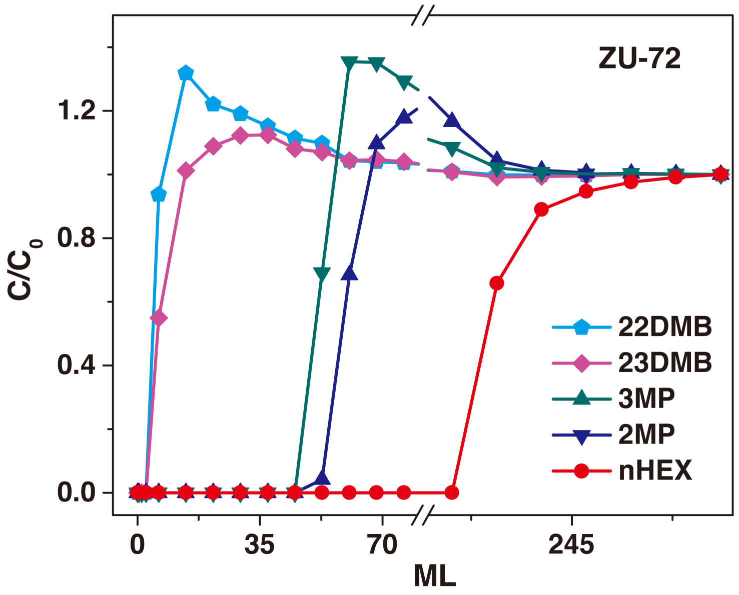

Supplementary Fig. 28. Experimental breakthrough curves for the equimolar mixture of nHEX/2MP/3MP/23DMB/22DMB on ZU-72 at 298 K. The horizontal axis represents the volume of the mixture of nitrogen gas and hexane isomers.

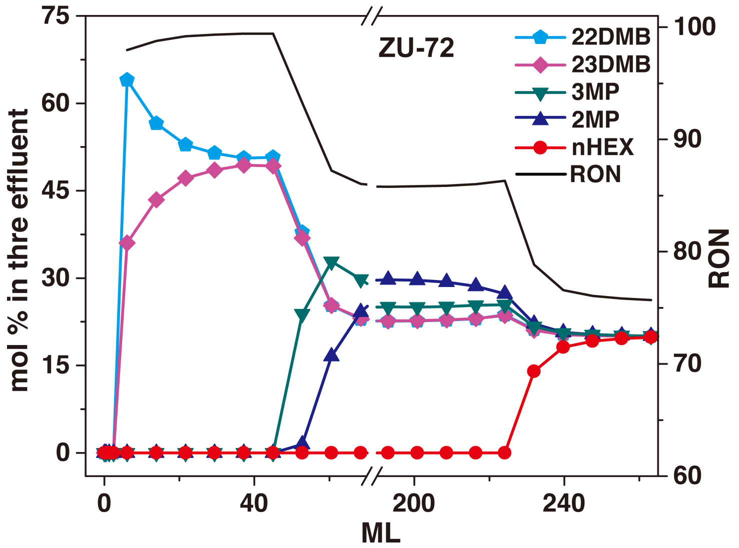

Supplementary Fig. 29. Experimental breakthrough curves for the equimolar mixture of nHEX/2MP/3MP/23DMB/22DMB on ZU-72 together with the RON calculated form the eluted mixture at 298 K. The horizontal axis represents the volume of the mixture of nitrogen gas and hexane isomers.

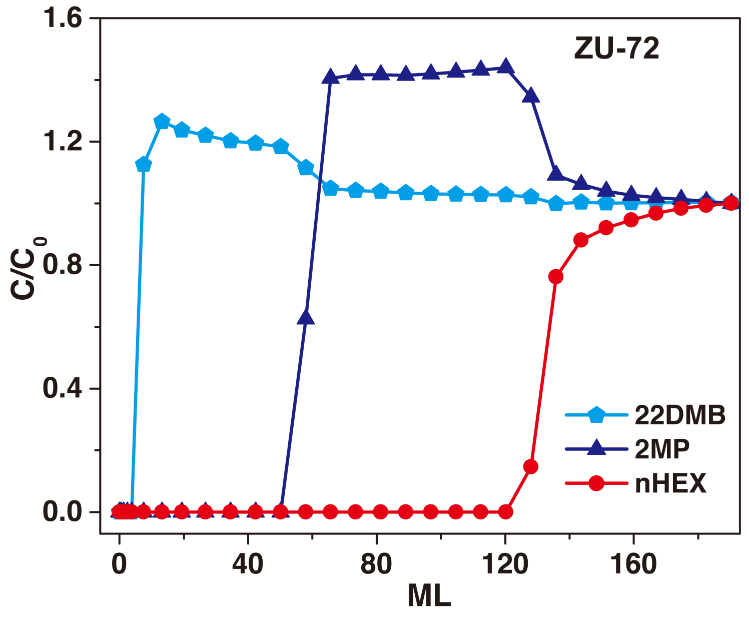

Supplementary Fig. 30. Experimental breakthrough curves for the equimolar mixture of nHEX/2MP/22DMB on ZU-72 at 298 K. The horizontal axis represents the volume of the mixture of nitrogen gas and hexane isomers.

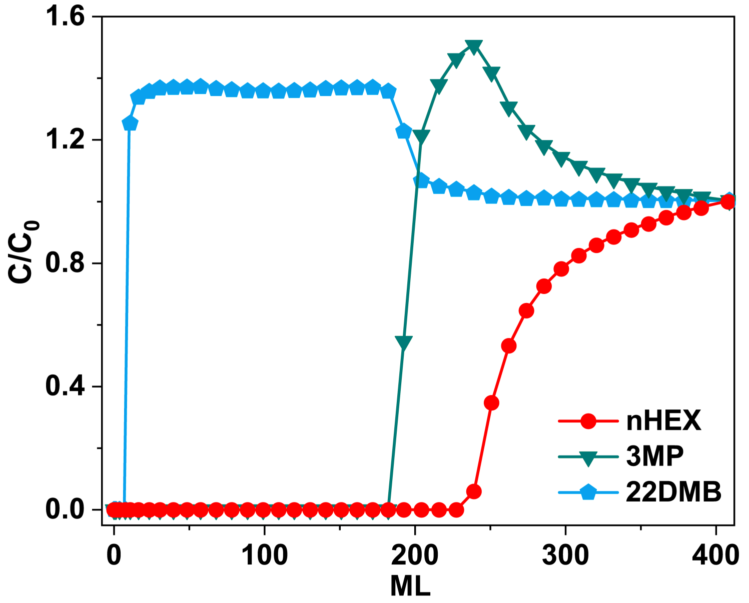

Supplementary Fig. 31. Experimental breakthrough curves for the equimolar mixture of nHEX/3MP/22DMB on SIFSIX-1-Cu at 298 K. The horizontal axis represents the volume of the mixture of nitrogen gas and hexane isomers.

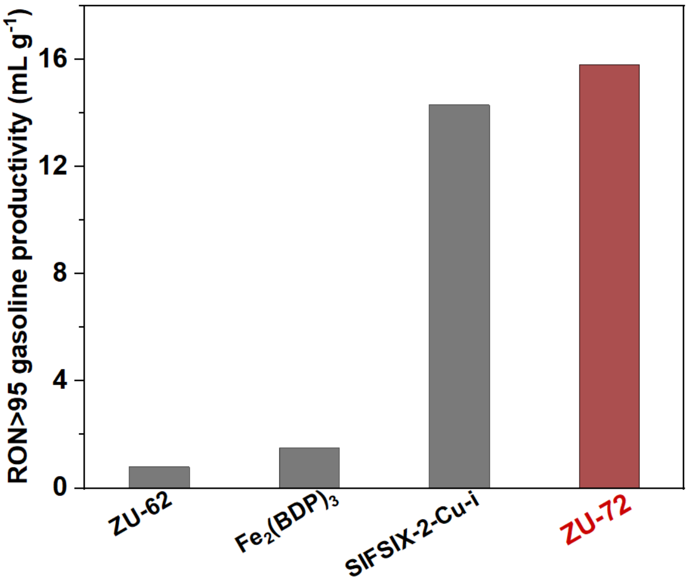

Supplementary Fig. 32. Comparison of the high quality gasoline (RON>95) productivity during the column breakthrough for the equimolar mixture of nHEX/2MP/3MP/23DMB/22DMB on ZU-72 with other good performing materials.

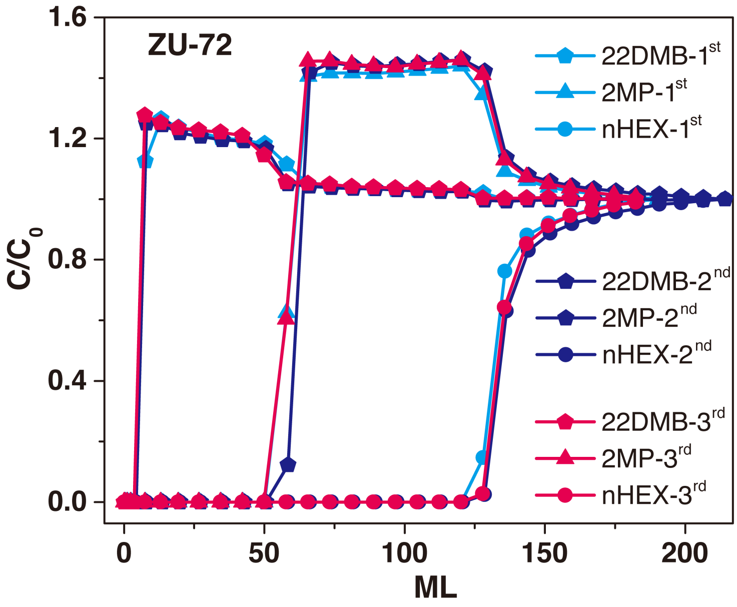

Supplementary Fig. 33. Cycling experimental breakthrough curves for the equimolar mixture of nHEX/2MP/22DMB on ZU-72 at 298 K. The horizontal axis represents the volume of the mixture of nitrogen gas and hexane isomers.

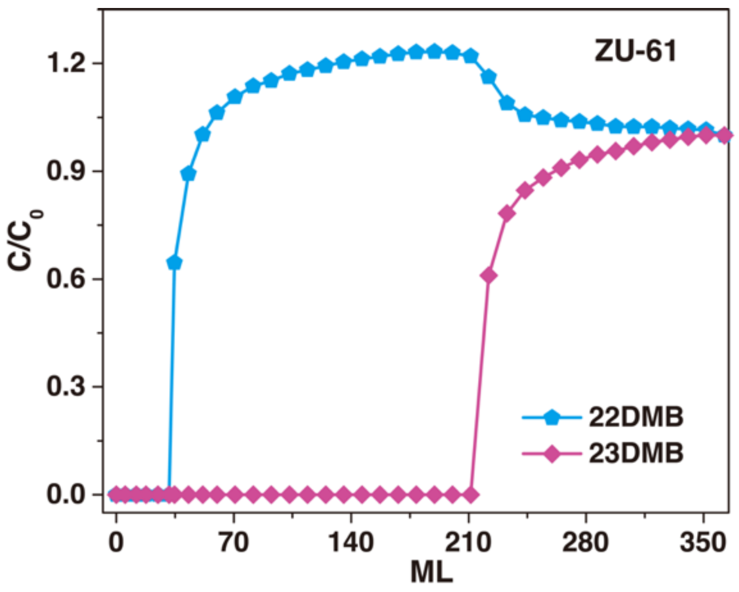

Supplementary Fig. 34. Experimental breakthrough curves for the equimolar mixture of 23DMB/22DMB on ZU-61 at 298 K. The horizontal axis represents the volume of the mixture of nitrogen gas and hexane isomers.

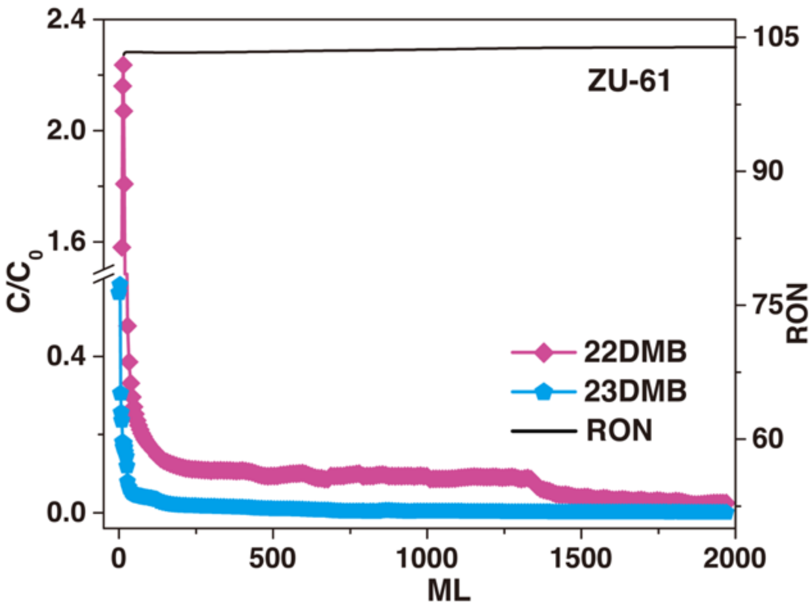

Supplementary Fig. 35. The desorption curves of 23DMB and 22DMB during the regeneration process under the nitrogen flow together with the RON calculated form the eluted mixture on ZU-61. The horizontal axis represents the volume of the mixture of nitrogen gas and hexane isomers.

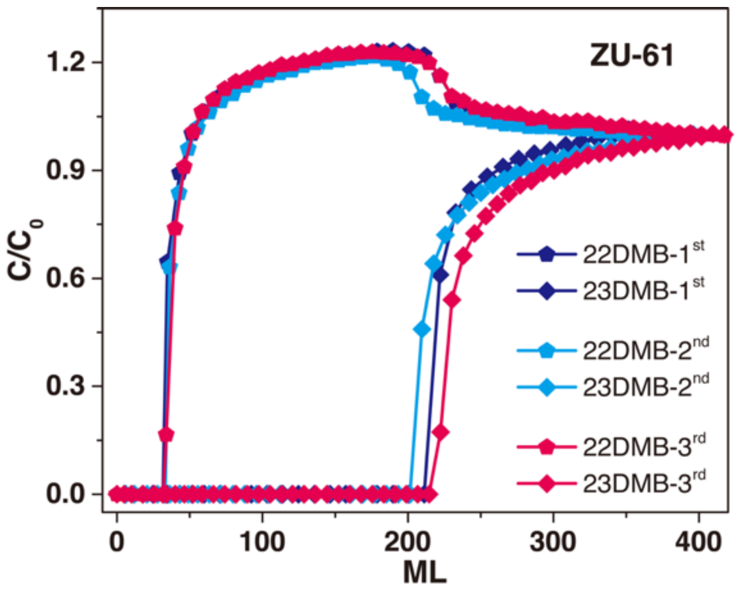

Supplementary Fig. 36. Cycling experimental breakthrough curves for the equimolar mixture of 23DMB/22DMB on ZU-61 at 298 K. The horizontal axis represents the volume of the mixture of nitrogen gas and hexane isomers.

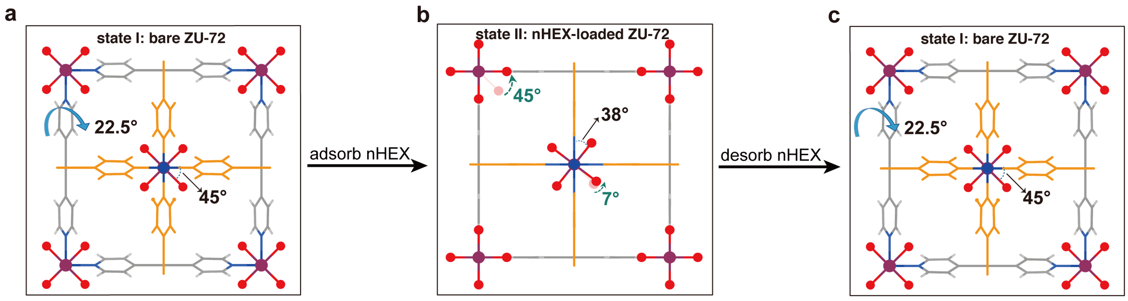

Supplementary Fig. 37. **Crystal Structures of ZU-72**. The activated structure of ZU-72 **(A)**. The structure of ZU-72 after loaded with nHEX molecules (**B**). The structure of ZU-72 after re-activated (**C**).

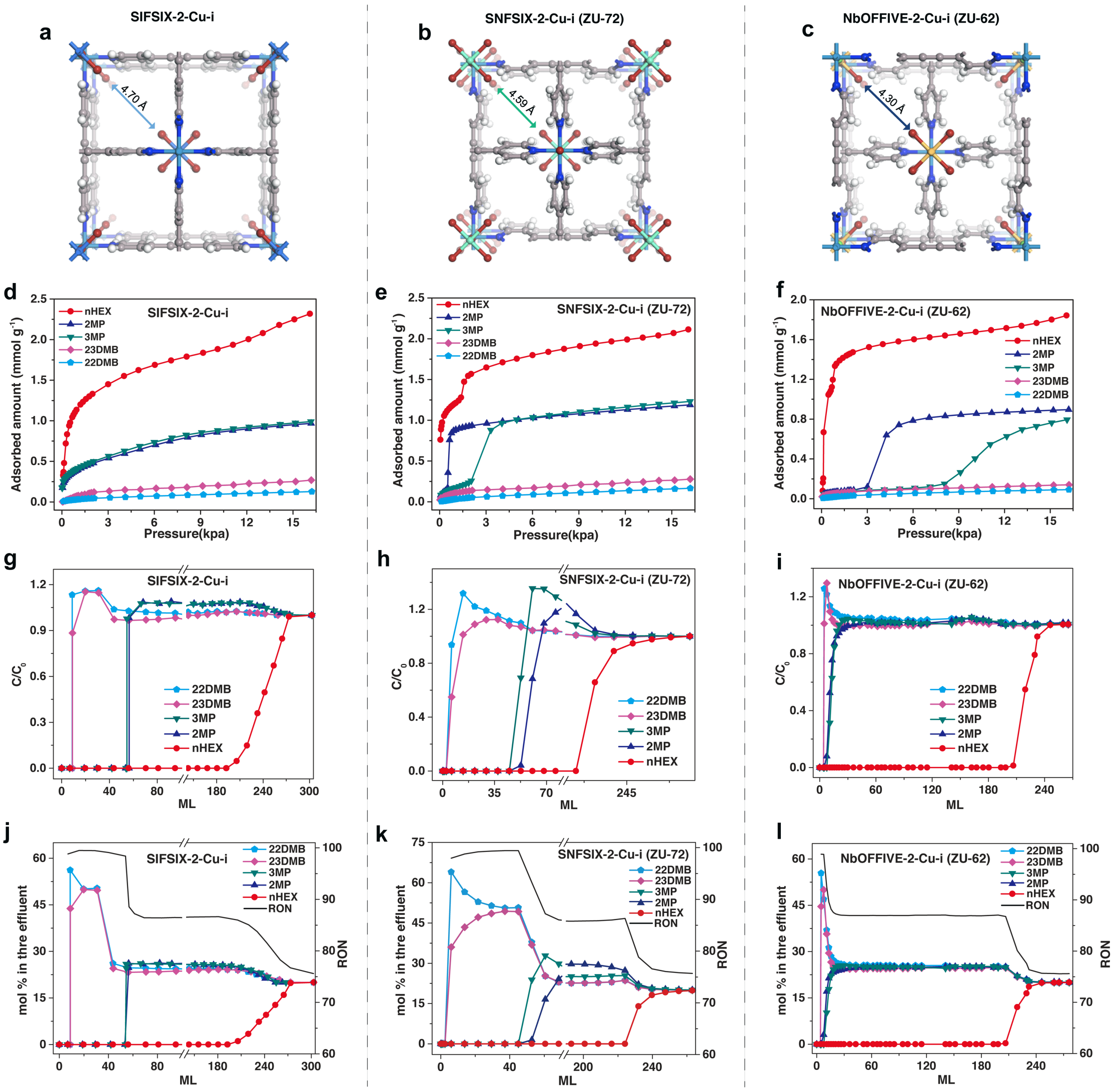

Supplementary Fig. 38. **The comparison of crystal structures and hexane isomers separation performance of varying anion functionalized MOFs**. The crystal structures of SiF_6^{2-}, SnF_6^{2-} and $NbOF_6^{2-}$ pillared MOFs, namely SIFSIX-2-Cu-i (a), ZU-72 (b) and ZU-62 (c), respectively. Pure-component vapor adsorption isotherms of n-hexane (nHEX), 2-methylpentane (2MP), 3-methylpentane (3MP), 2,3-dimethylbutane (23DMB) and 2,2-dimethylpentane (22DMB) on SIFSIX-2-Cu-i (d), ZU-72 (e) and ZU-62 (f), respectively at 298 K. Experimental breakthrough curves for the equimolar mixture of nHEX/2MP/3MP/23DMB/22DMB on SIFSIX-2-Cu-i (g), ZU-72 (h) and ZU-62 (i), respectively at 298 K. Experimental breakthrough curves for the equimolar mixture of nHEX/2MP/3MP/23DMB/22DMB on ZU-72 together with the RON calculated form the eluted mixture SIFSIX-2-Cu-i (j), ZU-72 (k) and ZU-62 (l), respectively at 298 K. The horizontal axis represents the volume of the mixture of nitrogen gas and hexane isomers (g-l).

Supplementary Tables

Supplementary Table 1. Comparison of the uptake and selectivity of hexane isomers on various materials for five-component isomers

	Temperature (K)	Pressure (kPa)	nHEX uptake (mmol g^{-1})	2MP uptake (mmol g^{-1})	3MP uptake (mmol g^{-1})	23DMB uptake (mmol g^{-1})	22DMB uptake (mmol g^{-1})	**Uptake ratio of MP/DMB**	Ref.
NU-2004	298	12	1.35	0.79	0.82	0.27	0.26	**3.04**	[1]
NU-2000	298	12	1.53	0.95	0.87	1.09	0.61	**1.07**	[1]
NU-2200	298	12	1.28	1.00	0.87	0.19	0.15	**5.50**	[2]
UU-200	303	12	1.67	1.23	1.12	0.37	0.1	**5.00**	[3]
$Fe_2(BPD)_3$	433	16	-	1.12	1.32	1.1	1.09	**1.11**	[4]
$Fe_2(BPD)_3$	433	12	1.28	1.04	1.16	0.92	0.92	**1.20**	[4]
Al-bttotb	303	16	1.74	1.15	1.06	0.42	0.06	**4.60**	[5]
Al-bttotb	303	12	1.71	1.13	1.04	0.41	0.05	**4.72**	[5]
ZU-62	298	12	1.71	0.87	0.62	0.15	0.11	**5.73**	**This work**
SIFSIX-2-Cu-i	298	12	1.98	1.11	1.05	0.25	0.14	**5.53**	**This work**
**ZU-72**	**298**	**16**	**2.12**	**1.19**	**1.23**	**0.26**	**0.15**	5.90	**This work**
**ZU-72**	**298**	**12**	**2**	**1.13**	**1.17**	**0.22**	**0.11**	6.97	**This work**

*MP is defined as (2MP + 3MP)/2;

*DMB is defined as (23DMB + 22DMB)/2

Supplementary Table 2. Comparison of the uptake and selectivity of hexane isomers on various materials

	Temper ature (K)	Press ure (kPa)	nHEX uptake (mmol g^{-1})	2MP uptake (mmol g^{-1})	3MP uptake (mmol g^{-1})	23DMB uptake (mmol g^{-1})	22DMB uptake (mmol g^{-1})	Uptake ratio of 23MB/22DMB	Ref.
$Ca(H_2tcpb)$	303	12	1.77	-	1.93	-	1.16	-	[6]
CAU-10-H/Br	303	12	1.57	-	0.75	-	0.12	-	[7]
MOF-1	313	16	0.41	-	0.08	-	0.06	-	[8]
MOF-1	313	12	0.36	-	0.06	-	0.05	-	[8]
$Zn_2(Hbdc)(dmtrz)_2$	298	16	1.5	-	1.3	-	0.23	-	[9]
$Zn_2(Hbdc)(dmtrz)_2$	298	12	1.43	-	1.26	-	0.2	-	[9]
MIL-53(Fe)-$(CF_3)_2$	313	16	0.32	-	0.29	-	0.23	-	[10]
MIL-53(Fe)-$(CF_3)_2$	313	12	0.3	-	0.27	-	0.21	-	[10]
HIAM-302	303	16	1.84	-	1.01	-	0.12	-	[11]
HIAM-302	303	12	1.66	-	0.94	-	0.11	-	[11]
HIAM-203	303	16	1.57		1.37		0.06	-	[12]
HIAM-203	303	12	1.55		1.36		0.05	-	[12]
Zn-tcpt	303	16	3.09		2.05		0.05		[13]
Zn-tcpt	303	12	2.95		2.02		0.04		[13]
UIO-66	303	12	2.66	-	2.66	2.65	-	-	[14]
Zr-abtc	303	12	2.49	-	2.07	1.1	-	-	[15]
1	298	12	0.86	-	0.63	-	0.46	-	[16]
Ni-Asp	303	12	1.50		0.22		0.16	-	[17]
MoOFOUR-Co-tpb	303	12	1.52		0.89		0.09	-	[18]
Mn-dhbq	303	12	1.81		1.80	0.93		-	[19]
CopzNi	303	12	2.13	1.37			0.07	-	[20]

HIAM-410	303	6.7	1.42		1.30		1.21	-	[21]
MFI	423	16	0.71	0.57	-	0.53	0.52	1.02	[22]
MFI	423	12	0.66	0.55	-	0.5	0.5	1.00	[22]
Zeolite BETA	423	16	0.82	-	0.81	0.74	0.7	1.16	[23]
Zeolite BETA	423	12	0.79	-	0.78	0.69	0.66	1.18	[23]
UU-200	303	12	1.67	1.23	1.12	0.37	0.1	3.70	[3]
$Fe_2(BPD)_3$	433	16	-	1.12	1.32	1.1	1.09	1.01	[4]
$Fe_2(BPD)_3$	433	12	1.28	1.04	1.16	0.92	0.92	1.00	[4]
Al-bttotb	303	16	1.74	1.15	1.06	0.42	0.06	7.00	[5]
Al-bttotb	303	12	1.71	1.13	1.04	0.41	0.05	8.20	[5]
ZU-62	298	12	1.71	0.87	0.62	0.15	0.11	1.36	This work
SIFSIX-2-Cu-i	298	12	1.98	1.11	1.05	0.25	0.14	1.79	This work
ZU-61	298	12				3.75	1.33	2.82	
**ZU-72**	**298**	**16**	**2.12**	**1.19**	**1.23**	**0.26**	**0.15**	1.73	**This work**
**ZU-72**	**298**	**12**	**2**	**1.13**	**1.17**	**0.22**	**0.11**	2.00	**This work**
**SIFSIX-1-Cu**	298	16	**5.07**		**4.47**	4.25	0.21	**20.23**	**This work**
**SIFSIX-1-Cu**	298	12	**4.98**		**4.42**	4.21	0.19	**22.16**	**This work**

Supplementary Table 3. Crystal Structure data and refinement conditions for solvent-loaded ZU-72

Unit cell parameters	
Formula sum	C24 H16 Cu F6 N4 Sn
Formula weight	656.64 g/mol
Crystal system	tetragonal
Space –group	I 4/m m m (139)
Cell parameters	a=13.7966(17) Å c=8.4472(10) Å
Cell ratio	a/b=1.0000 b/c=1.6333 c/a=0.6123
Cell volume	1607.89(40) $Å^3$
Z	2
Calc.density	1.3562 g/cm^3

Supplementary Table 4. Crystal Structure data and refinement conditions for activated ZU-72

Unit cell parameters	
Formula sum	C24 H16 Cu F6 N4 Sn
Formula weight	656.64 g/mol
Crystal system	tetragonal
Space –group	I 4/m m m (139)
Cell parameters	a=13.713(3) Å c=8.3434(19) Å
Cell ratio	a/b=1.0000 b/c=1.6436 c/a=0.6084
Cell volume	1568.95(70) $Å^3$
Z	2
Calc.density	1.386986 g/cm^3

Supplementary Table 5. Crystal Structure data and refinement conditions for nHEX-loaded ZU-72

Unit cell parameters	
Formula sum	C24 H16 Cu F6 N4 Sn
Formula weight	656.64 g/mol
Crystal system	tetragonal
Space –group	I 4/m m m (139)
Cell parameters	a=13.7095(8) Å c=8.3735(6) Å
Cell ratio	a/b=1.0000 b/c=1.6372 c/a=0.6108
Cell volume	1573.8(2) $Å^3$
Z	2
Calc.density	1.38558 g/cm^3

Supplementary Table 6. Crystal Structure data and refinement conditions for re-activated the ZU-72 loaded with nHEX

Unit cell parameters	
Formula sum	C24 H16 Cu F6 N4 Sn
Formula weight	656.64 g/mol
Crystal system	tetragonal
Space –group	I 4/m m m (139)
Cell parameters	a=13.744(3) Å c=8.3477(19) Å
Cell ratio	a/b=1.0000 b/c=1.6464 c/a=0.6074
Cell volume	1576.86(70) $Å^3$
Z	2
Calc.density	1.38289 g/cm^3

Supplementary References

1. Idrees, K. B. et al. Robust Carborane-Based Metal−Organic Frameworks for Hexane Separation. *J. Am. Chem. Soc.* **145**, 23433-23441 (2023).

2. Lal, B. et al. Pore aperture control toward size-exclusion-based hydrocarbon separations. *Angew. Chem. Int. Ed.* **62**, e202219053 (2023).

3. Zhang, Z. Q., Peh, S. B., Kang, C. J., Yu, K. X., Zhao, D. Efficient splitting of alkane isomers by a bismuth-based metal-organic framework with auxetic reentrant pore structures. *Angew. Chem. Int. Ed.* **61**, e20221180 (2022).

4. Herm, Z. R. et al. Separation of hexane isomers in a metal-organic framework with triangular channels. *Science* **340**, 960-964 (2013).

5. Yu, L. et al. Splitting mono- and dibranched alkane isomers by a robust aluminum-based metal−organic framework material with optimal pore dimensions. *J. Am. Chem. Soc.* **142**, 6925-6929 (2020).

6. Wang, H. et al. One-of-A-Kind: A microporous metal-organic framework capable of adsorptive separation of linear, mono- and di-branched alkane isomers via temperature- and adsorbate-dependent molecular sieving. *Energy Environ. Sci.*, **11**, 1226-1231 (2018).

7. Yu, Q. C. et al. A pore-engineered metal-organic framework with mixed ligands enabling highly efficient separation of hexane isomers for gasoline upgrading. *Sep. Purif. Technol.*, **268**, 118646 (2021).

8. Ba´rcia, P. S., Zapata, F., Silva, J. A. C., Rodrigues, A. E., Chen, B. L. Kinetic separation of hexane isomers by fixed-bed adsorption with a microporous metal-organic framework. *J. Phys. Chem. Lett.*, **111**, 6101-6103, (2007).

9. Ling, Y. et al. A zinc(II) metal–organic framework based on triazole and dicarboxylate ligands for selective adsorption of hexane isomers. *Chem. Commun*., **47**, 7197-7199 (2011).
10. Mendes, P. A. P. et al. A complete separation of hexane isomers by a functionalized flexible metal organic framework. *Adv. Funct. Mater.,* **24**, 7666-7673 (2014).
11. Yu, L. et al. A microporous metal−organic framework incorporating both primary and secondary building units for splitting alkane isomers. *J. Am. Chem. Soc.,* **144**, 3766-3770 (2021).
12. Lin, Y. H. et al. Temperature-programmed separation of hexane isomers by porous calcium chloranilate metal-organic framework. *Angew. Chem. Int. Ed.,* **61**, e202214060 (2022).
13. Yu, L. et al. High-capacity splitting of mono- and dibranched hexane isomers by a robust zinc-based metal–organic framework. *Angew. Chem. Int. Ed.,* **134**, e202211359 (2022).
14. Barcia, P. S. et al. Reverse shape selectivity in the adsorption of hexane and xylene isomers in MOF UiO-66. *Microporous Mesoporous Mater*., **139**, 67-73 (2011).
15. Wang, H. et al. Topologically guided tuning of Zr-MOF pore structures for highly selective separation of C6 alkane isomers. *Nat Commun*. **9**, 1745 (2018).
16. Lv, D. F. et al. Iron-based metal−organic framework with hydrophobic quadrilateral channels for highly selective separation of hexane isomers. *ACS Appl. Mater. Interfaces.,* **10**, 6031−6038 (2018).
17. Chen, R. D. et al. Robust Nickel Aspartate Framework for Shape Recognition of Hexane Isomers. *ACS Sustainable Chem. Eng*., **10**, 11330-11337 (2022).
18. Su, Y. et al. Dual pore-size sieving in a novel oxygenate-pillared microporous adsorbent for C6 alkane isomers separation. *AIChE J.* **69**, e17937 (2023).
19. Chen, R. D. et al. Sequential Separation of Linear, Mono-, and Di-Branched Hexane Isomers on a Robust Coordination Polymer with Nonbonding Flexibility. *Small.*, **19**, 2207367 (2023).
20. Zheng, F, et al. Temperature-swing molecular exclusion separation of hexane isomers in robust MOFs with double-accessible open metal sites. *Chem Eng J.*, **460** 141743 (2023).
21. Guo, F. A.et al. Linker Vacancy Engineering of a Robust ftw-type Zr-MOF for Hexane Isomers Separation. *Angew. Chem. Int. Ed.*, **62**, e202303527 (2023).
22. Ferreira, A. F. P., Hazeleger · Alfred Bliek, M. C. M. Can alkane isomers be separated? Adsorption equilibrium and kinetic data for hexane isomers and their binary mixtures on MFI. *Adsorption*., **13**, 105-114 (2007).
23. Barcia, P. S., Silva, J. A. C., Rodrigues, A. E. Separation by Fixed-Bed Adsorption of Hexane Isomers in Zeolite BETA Pellets. *Ind. Eng. Chem. Res*., **45**, 4316-4328 (2006).
